# Writing Technical Reports for Simulation in Education for Health Professionals: Suggested Guidelines

**DOI:** 10.7759/cureus.371

**Published:** 2015-11-02

**Authors:** Adam Dubrowski, Sabrina Alani, Tina Bankovic, Andrea Crowe, Megan Pollard

**Affiliations:** 1 Emergency Medicine, Pediatrics, Memorial University of Newfoundland; 2 Marine Institute, Memorial University of Newfoundland; 3 Emergency Medicine, Memorial University of Newfoundland; 4 Oncology, Memorial University of Newfoundland; 5 Clinical Learning and Simulation Centre, Memorial University of Newfoundland; 6 Faculty of Nursing, Memorial University of Newfoundland

**Keywords:** simulation based medical education, simulation, guidelines

## Abstract

Simulation is an important training tool used in a variety of influential fields. However, development of simulation scenarios - the key component of simulation – occurs in isolation; sharing of scenarios is almost non-existent. This can make simulation use a costly task in terms of the resources and time and the possible redundancy of efforts. To alleviate these issues, the goal is to strive for an open communication of practice (CoP) surrounding simulation. To facilitate this goal, this report describes a set of guidelines for writing technical reports about simulation use for educating health professionals. Using an accepted set of guidelines will allow for homogeneity when building simulation scenarios and facilitate open sharing among simulation users. In addition to optimizing simulation efforts in institutions that are currently using simulation as an educational tool, the development of such a repository may have direct implications on developing countries, where simulation is only starting to be used systematically. Our project facilitates equivalent and global access to information, knowledge, and highest-caliber education - in this context, simulation – collectively, the building blocks of optimal healthcare.

## Introduction

Simulation, defined as a replication of a task or an event for the purpose of training and/or assessment [1] in fields ranging from medical and health professions to military, to business, to off-shore industries, is an expensive proposition [1]. The costs are related to the initial set-up of facilities and infrastructure, operations, and programming [2]. One major resource-draining, and often overlooked, activity relates to the process of developing simulation scenarios – a fundamental element of simulation. The scenarios are scripts that transform physical simulators from passive objects to educational tools by incorporating learning objectives, experiences, pre-briefing, briefing and de-briefing strategies, and assessment metrics. The scenarios can be very simple, incorporating basic simulators designed to teach fundamental skills to individual learners, to very complex, incorporating computerized mannequins, standardized patients, and interprofessional teams. 

To date, simple and complex scenarios alike are developed at the level of the individual educator—for example, a doctor who wants to teach his/her students specific skills. These scenarios exist as part of a larger curriculum, and at best are passed along from year to year in the form of course materials.

Because simulation has not traditionally been part of education and training practice, it is often seen as an additional and resource-demanding task and, therefore, may not be readily accepted into existing curriculums. In addition, simulation scenarios reside in local curricular repositories and course descriptions. This translates into large-scale redundancy of efforts, time, and money, as well as heterogeneity of quality of scenarios across the programs. For example, learning the technique of intubating a critically sick patient using a simulator is the same whether this is done in North America, Europe, or Asia, yet sharing of simulation scenarios is virtually non-existent.

The concept of a community of practice (CoP) may be useful in synchronizing system-wide efforts. The term CoP is of relatively recent coinage, even though the phenomenon it refers to is age-old. In its basic form, a CoP is a collection of people who work together towards a common goal sharing similar processes [3]. In the past year, using the open nature of Cureus.com, we have attempted to develop a simulation community of practice and an open repository platform for simulation cases [3-5]. The pilot phase resulted in six simulation cases published from both medical and nursing education [6-11]. These cases have been peer-reviewed by experts in the field, refined based on feedback, published and are now available for anyone in the world to access them. Although thematically different, all cases follow a similar pattern, which was refined iteratively based on the reviewers’ comments.

The purpose of this technical report is to provide the readers, future contributors, and, hopefully, members of the medical community with a set of guidelines of what elements should be included when developing simulation scenarios for open sharing. By definition, following guidelines is never mandatory, and therefore, these guidelines aim to streamline the process of simulation case creation in order to standardize the presentation and content.

## Technical report

**Figure 1:**
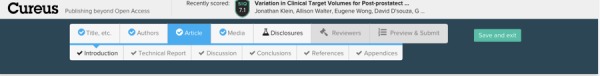
The Four Elements of a Technical Report, As They Currently Appear in Cureus.

Each technical report should incorporate the majority of the applicable nine elements described below. These elements are designed to help the author, future reader, and educator who use simulation to understand the context (such as the learner and environment), the required resources, scenario logic, learning objectives, and anticipated outcomes of the case. Figure 1 depicts how these four elements translate to the current technical report layout. 

### Element 1: Introduction to the technical report

The introduction to the scenario should briefly describe four aspects of the simulation: context, inputs, process, and outcomes or products [12].  These four aspects should be written such that the potential reader can quickly assess the applicability of this particular scenario to their own context, the resources and processes that are available, and potential outcomes. 

*Context:* What is the educational setting in which this simulation is taking place? Is there any specific institutional, political, or other context that needs to be understood? What skills are being learned (in general terms, e.g. technical, nontechnical, inter-professional, etc.)?

*Inputs:* What is needed in terms of equipment, technology, and operators? What level and type of learners (physicians, nurses, etc.) are involved?

*Process:* How is the case delivered? What are the educational/pedagogical underpinnings of this simulation? How are the learners prepared for the simulation? What are the feedback/debriefing protocols?

*Outcome:* What was, should, or could be measured? Are there any specific and validated assessment metrics/instruments that could be applied to this simulation to look for effectiveness?

*Objectives:* What are the learning objectives? These should be listed as primary, secondary, tertiary, and so forth.

### Element 2: Technical report - participants

The authors should describe in detail all those who will participate in the simulation event. Special attention should be paid to the learners, with detailed description of their level of training, profession, and any other special skills that describe their population. If confederates (trained actors) or other professionals (for example, nurses and respiratory therapists) are used as active participants, they and their roles should also be described in detail.

### Element 3: Technical report - setting and equipment

The description of the setting should focus on the essentials of the location. For example, is the simulation taking place as an in-situ simulation in a hospital, or does it happen in a simulation laboratory? What type of model/simulator is being used, and what type of other specialized equipment needs to be in the room in order to re-create the required context? Finally, are there any special moulage requirements to be used during the simulation event to achieve the required levels of realism?

### Element 4: Technical report - scenario template

The scenario template should describe the scenario’s progress, from the pre-simulation briefing to the possible ways in which the simulation can progress. The template should also explain the scenario’s instructional design.

*Briefing*: Authors should describe the methods in which the learner and the educators were briefed before simulation.

*Instructional design*: In general terms, authors should convey how instructional variables, such as task variability, a range of difficulty, the importance of sequence, and the introduction of stress, will be moderated.

**Figure 2:**
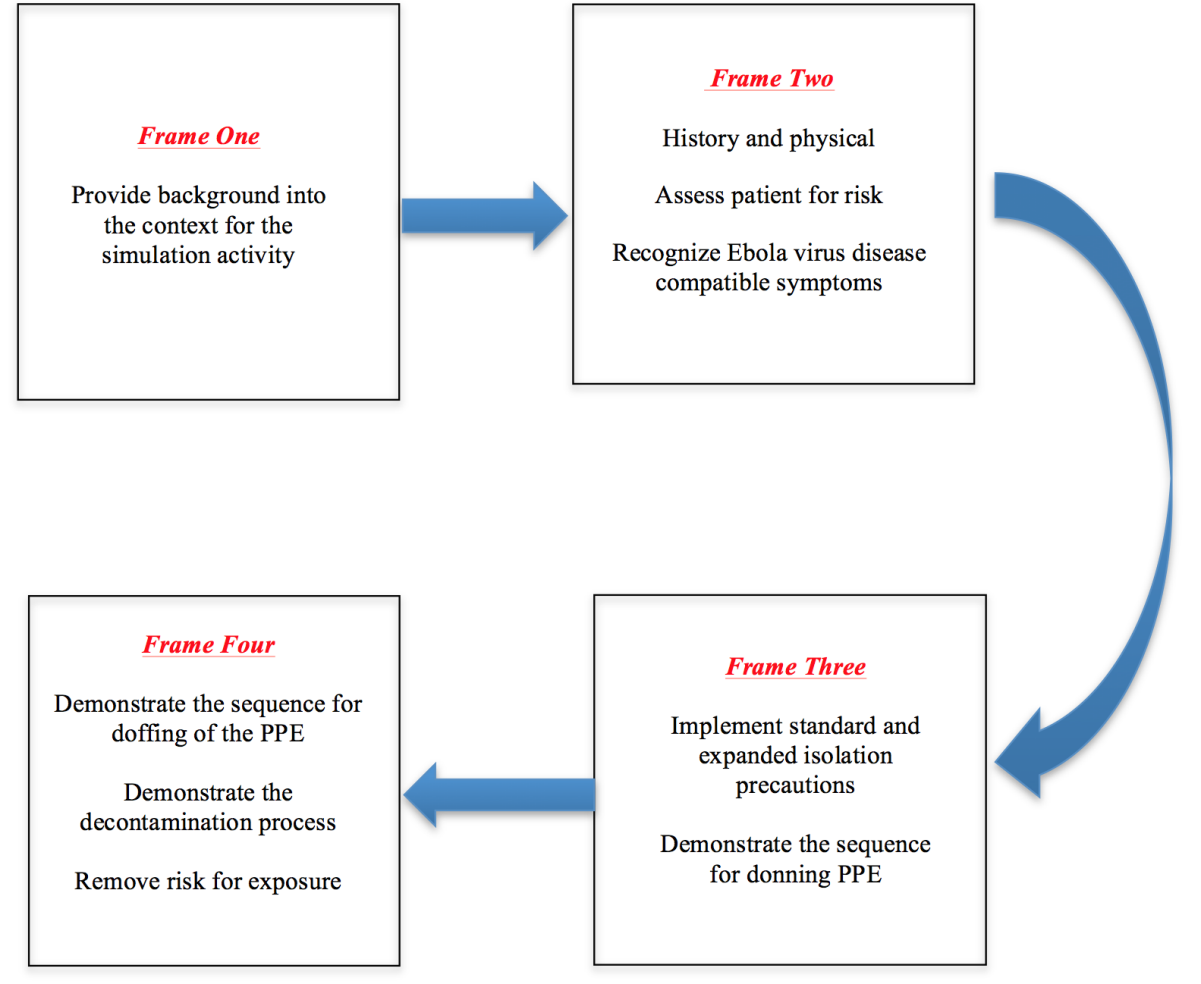
Simulated Ebola Case Scenario Frames As seen in http://www.cureus.com/articles/2704-ebola-virus-hemorrhagic-fever-a-simulation-based-clinical-education-experience---designed-for-senior-undergraduate-nursing-students

There are many ways in which the scenario’s evolution can be presented. Tables and figures, such as design templates, are most informative for quick referencing. For example, Manuel, et al. have used a figure to document changes from one frame of a scenario to another (Figure 2) [11]. Each frame was defined as part of a scenario linked to specific learning objectives, and for each frame, the authors provide a list of expected responses and actions. For incorrect responses and actions, they also provide a table with tightly-linked hints and prompts to guide the learner towards the expected responses and action.  

**Figure 3:**
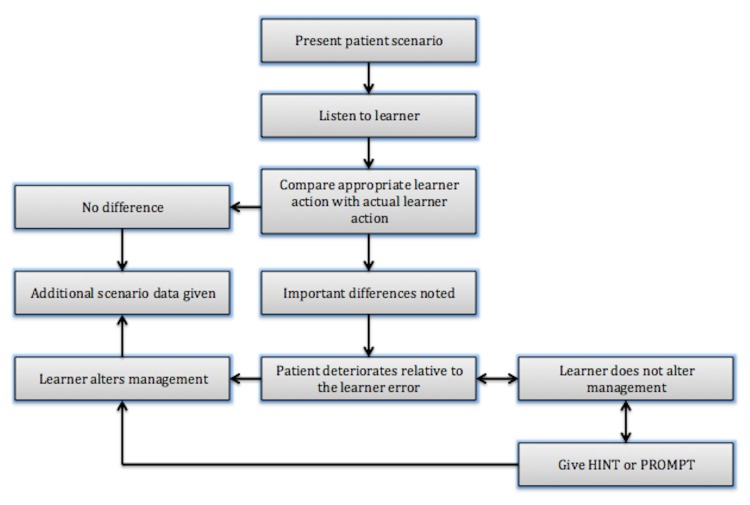
Algorithm Adapted as a Script to Show Potential Events in a Simulation Session As seen in http://www.cureus.com/articles/2643-pregnancy-and-privacy-in-an-emergency-department-a-simulated-session

Similarly, Black, et al. use a figure to depict an “if and then” algorithm formulated as a detailed and precise stepwise script (Figure 3) [8]. Like Manuel, et al.’s figure, these authors use hints to guide the learner towards expected responses and actions [11].

The alternative method of presenting a scenario is a table. Angus, et al. use a table to advance through the scenario [6]. The use of tables for scenarios that contain a lot of patient status information, such as vital signs, may be advantageous. For a good example of a table used this way, please refer to reference 6 (published on the Cureus website). 

Both figures and tables need to have a number of critical components to make them most useful for the potential users. They need to list learning objectives and tie these objectives to specific responses and actions. Both need to provide a list of what is considered a correct response and action, as well as hints on to how to deal with incorrect responses and actions. These are termed as points of possible deterioration where, if the educators observe incorrect responses and actions, they may choose to provide hints and correct the course of the scenario, or allow the scenario (and the patient) to deteriorate. Therefore, both figures and tables should follow a deteriorating patient scenario (DPS) framework [13]. In its original conception, DPS is a low-fidelity simulation activity that provides learners with opportunities for deliberate practice and feedback. The educator takes on the role of a patient with deteriorating vital signs. Learners have to decide what steps to take in treating or stabilizing the patient, and the case evolves in response to their actions. Throughout the activity, learners get the opportunity to compare their thinking to an expert as well as to learners that are more senior. During the debriefing, learners are supported by the educator in reflection on their actions during the scenario. Although the utility of DPS framework to different simulation modalities may vary, some constructs of objectives, experimentation, and feedback are readily transferrable across all simulation modalities and should be included in figures and tables describing any simulation scenario.

The essential components of any figure or table describing a simulation scenario are as follows:

1) Frame

2) Objective as related to the frame related to this frame (required)

3) Points of possible deterioration (required)

4) Hints/feedback (if applicable)

5) Other (if applicable)

### Element 5: Technical report - supplementary materials

Frequently, authors will make references to supplementary information that the trainees may require. This information could include ECG, EEG readings, screenshots of vital signs monitors, blood work, and other laboratory tests. Whenever possible, these should be presented as figures in the manuscript and referenced in the general description of the case, under the “Case” heading, as well as within the scenario template for ease of referencing. For example, Whalen, et al. uses a JPG of an electrocardiogram (EKG or ECG) demonstrating sinus bradycardia typically seen in a beta-blocker overdosed patient that was described in their template [7]. 

In addition, video files may be very useful supplementary materials.  In this case, the videos can serve two purposes. First, like figures, they may offer additional information or tests that would be requested by the trainee. Black, et al. used vignettes of ultrasound videos taken from real patients to illustrate various views of the uterus with the bladder-uterine juxtaposition that was critical to their case (NOTE: Authors must obtain patient consent to use their videos) (8). The second purpose of including videos is to illustrate what a simulation scenario would look like. Some simulations can take place in unique settings, or follow a unique discourse, which is best illustrated with a video clip. Renouf, et al. made great use of a short YouTube video clip demonstrating learning objectives as well as incorrect and correct interactions with the difficult standardized patient, which was the main purpose of their simulation [9].

### Element 6: Technical report - feedback and debriefing

Next to practice itself, feedback and debriefing are the most important educational features of simulation [14]. This section should focus on describing the methods in which the learners and the educators were debriefed after the simulation and/or how feedback was provided during teaching. Fanning and Gaba [15] provide a great overview of styles and methods, and this article may be a good starting point to exploring the relevant descriptions [1].

### Element 7: Technical report - assessments (if applicable)

Similar to debriefing methods, the authors are encouraged to provide assessment tools, or references to these tools, with specific attention to documentation of their validity and reliability. In addition to the assessment of learners, the authors should consider providing methods and assessment tools for the assessment of skills of the debriefers.

### Element 8: Summary

Authors should re-emphasize the purpose, context, inputs, process, and outcomes/products. If possible, the authors are encouraged to comment on aspects of the simulation event that could be generalized to other contexts, as well as unanticipated outcomes and lessons learned [2, 16].

### Element 9: Conclusions

Each report should finish with a general statement about why simulation is a useful tool to train the particular set of skills, with possible speculation of other types of simulations or educational approaches that could supplement or replace simulation. Economic factors, such as costs and access to simulators, could be considered [2].

## Discussion

The purpose of this technical report was to provide the readers, future contributors, and educators who use simulation as a teaching modality with a set of guidelines of the elements to include when developing simulation scenarios for open sharing. Based on our experience to date, each technical report should incorporate nine elements (Introduction, Participants, Setting and Equipment, Scenario Template, Supplementary Equipment, Feedback and Debriefing, Assessment, Summary, and Conclusions) designed to help the author, future reader, and educator using simulation to understand the context (such as learner and environment), required resources, scenario logic, learning objectives, and anticipated outcomes of the simulation scenario described.

## Conclusions

Global access to information and knowledge, as well as highest-caliber education - in this context, simulation - requires sustainable access to new learning technologies, resources, and techniques in both developed and developing countries and regions.

The purpose of this technical report was to provide a set of guidelines to write and publish simulation scenarios. The ultimate goal of this project is to use open publishing to build a repository of shareable, peer-reviewed simulation scenarios that can be submitted and accessed anywhere in the world. This approach allows the developers of the scenarios to claim scholarly credit for their work, it benefits the quality of the scenarios by introduction of peer-review and crowd-based rating, and ultimately, it adheres to the tenants of global access of information and knowledge.
